# Effect of fencing on regional ecological networks in the northern Tibetan Plateau

**DOI:** 10.3389/fpls.2023.1051881

**Published:** 2023-01-31

**Authors:** Yifei Zhao, Shiliang Liu, Yuhong Dong, Fangfang Wang, Hua Liu, Yixuan Liu

**Affiliations:** ^1^ State Key Laboratory of Water Environment Simulation, School of Environment, Beijing Normal University, Beijing, China; ^2^ Research Institute of Forestry, Chinese Academy of Forestry, Beijing, China

**Keywords:** fencing, grazing, land use change, ecological network, circuit theory, the Qinghai-Tibet plateau

## Abstract

Fencing is an essential measure for the rehabilitation and conservation of grasslands on the Qinghai-Tibet Plateau. However, its construction could change the distribution and migration of wildlife, thus affecting the integrity of the ecological networks for local wild animal movement. It is of great significance to quantify the potential impact of fencing on ecological network connectivity associated with land-use changes at regional scale. In this study, taking the northern Tibetan Plateau as the study area, we explored the ecological network change using circuit theory approach under different scenarios at county scale. Among them, this study set up four different scenarios according to the economic growth rate, population growth rate and the sustainable development of society and environment. The results showed that: 1) with increased grazing intensity and enhanced human activities from 1990 to 2015, the grasslands of the northern Tibetan Plateau were greatly degraded, most of which was converted into the barren land, and the conversion proportion was as high as 90.84%, which lead to a decreasing trend of the current density of ecological network in most counties and deterioration of ecological connectivity; 2) fencing construction has reduced regional current density, while fencing intensity is positively correlated with current density loss at county scale. Among them, the counties with serious current density loss were distributed in the northwest and southeast regions. The maximum loss ratio is 39.23%; 3) under four different future land use scenarios, coordinated economic, social and environmental development will have a positive effect on the ecological network. The results of the study have important ecological significance for developing reasonable conservation measures for grassland restoration, protecting wildlife, and maintaining regional ecological balance.

## 1 Introduction

The ecological challenges faced by grasslands are increasing due to intensified overgrazing pressure and continued climate warming (Li et al., 2018). With the aim of effective management of pasture resources, many pastoral regions have implemented fencing measures (Dawa, 2009). Fencing is the use of closed management of grass, prohibiting mowing and grazing, which can reduce undesirable human interference, create conditions for the recovery and regeneration of plants, and allow pasture to recuperate (Jurburg et al., 2017). Studies have shown that it allows the height, cover, biomass, and biodiversity of plant communities to increase (Zhao et al., 2019), while the soil fertility could be overall improved with the number of years of fencing (Manaye, 2017; Ping and Wang, 2018; Xu et al., 2020). However, while obtaining the benefits of grassland restoration and management, fence construction has become an obstacle to the movement of wild animals, which limits their normal activities such as foraging and resource acquisition, reducing the habitat area, and increasing habitat fragmentation. Some studies showed that the 12 years of fencing significantly increased vegetation cover, height, Richness index, and above and belowground biomass (Zhu et al., 2016). However, because of lacking of human interference, the fierce competition of plants, caused by limited resources, may induce intense Intra and interspecific competition in exclosure grasslands (Catford et al., 2012). This may lead to a loss of biodiversity and ultimately cause degradation of the ecosystem after long-term fencing (Su et al., 2015). In Africa, Percival (Sun et al., 2020a) has found that most wildlife deaths were caused by fence entanglements. Thus, fencing construction has a certain influence on multiple scales including biological mobility (Moseby et al., 2020), landscape connectivity (Andrew et al., 2018), and integrity of the regional ecological network (You et al., 2013).

The integrity of ecological network is a state in which landscape structure, ecological process, and function are organically combined (Wei et al., 2022), forming a composite system with complete ecological connectivity at regional scale (Liu et al., 2017). Designing an ecological network is of great significance for protecting biodiversity, maintaining ecosystem stability, and improving landscape connectivity (Nie et al., 2021). However, human activities such as land use change, agriculture and animal husbandry, as well as climate change, have a huge impact on the ecological network of grassland areas (Huang et al., 2021; Shen et al., 2021). High-intensity land development and utilization activities have resulted in the degradation of the ecological environment in some regions, the loss of species habitat, and the degree of landscape fragmentation seriously affects the migration and communication of species (Mu et al., 2021), leading to the reduction of biodiversity, which is related to human well-being (Song et al., 2021). Quantifying the ecological network change can effectively identify the internal mechanism of the ecological pattern and process under disturbance stress (Jodi and Aaron, 2021), which is not only conducive to maintaining regional ecological functions, promoting energy flow, and optimizing regional landscape patterns. It can also effectively deal with the fragmentation of wildlife habitats and the segmentation of ecological corridors (Modica et al., 2021). There are many methods to construct the ecological network, such as minimum cumulative resistance model, circuit theory and so on. In this study, circuit theory that simulates the difficulty of species crossing a resistance surface is selected for ecological network construction and analysis. The model can show the distribution and migration of species and present the ecological network in the region (Wei et al., 2022). In addition, compared with the minimum cumulative resistance model method, it can identify alternative path results that species may pass through when moving between ecological sources, which are not the least cost path, providing a supplement to the least cost path results generated by the minimum cumulative resistance model (Huang et al., 2021).At present, many studies related to ecological networks focused on borders (Pokorny et al., 2017), highways (Olsson and Widen, 2008), and nature reserves (Linnell, 2016). However, there are few relevant studies on the plateau where there is a lack of research data. Given the ecological importance of the plateau, a comprehensive study is needed to quantify its ecological network changes, especially under the climate change, land use change and fence construction.

The Qinghai-Tibet Plateau has a large area of natural grassland and abundant wildlife resources, which is an important ecological barrier in China (Sun et al., 2021). Due to its arid, high-altitude, and cold environment, it is extremely sensitive to the impact of human activities (Sun et al., 2020b; Li et al., 2021). The northern Tibetan Plateau, with a higher altitude, is more fragile (Piao et al., 2019), which is also seriously affected by human activities, and overgrazing (Zhang et al., 2019b). Remote sensing monitoring results showed that by 2010, the proportion of degraded grassland area in the northern Tibetan Plateau reached 58.2%, and the overall level was close to moderate degradation. Compared with the 1980s, the area of severely degraded and extremely degraded grassland in the northern Tibetan Plateau has increased, and the grassland degradation cannot be ignored. To solve the urgent problem of grassland degradation, large-scale fencing has been constructed in the northern Tibetan Plateau since 2004. Although fencing has a certain positive effect on grassland restoration (Yu et al., 2016), it also has some negative effects. However, there are few studies on its ecological externalities (Ito et al., 2010), such as creating obstacles for wildlife migration, ecosystem fragmentation, reduced landscape connectivity, and other negative issues at large scale (Cew et al., 2021). Moreover, previous studies found that land use in the northern Tibetan Plateau continues to change (Zhang et al., 2019a), while there are few related studies on the impact of land use change on the ecological network.

Given these issues, constructing the ecological network and exploring the impact of the fence on the ecological network of the northern Tibetan Plateau are urgently needed for regional biodiversity conservation and landscape connectivity improvement. In this study, taking the northern Tibetan Plateau as the research area with intensive fencing measures, we analyzed the impact of fencing on the ecological network associated with the historical land-use change and future scenarios using circuit theory at the county scale. The objectives were to: 1) explore the impact of fencing on the regional ecological network in the northern Tibetan Plateau; 2) analyze the changes in a regional ecological network under different land-use scenarios.

## 2 Materials and methods

### 2.1 Study area

**Figure 1:**
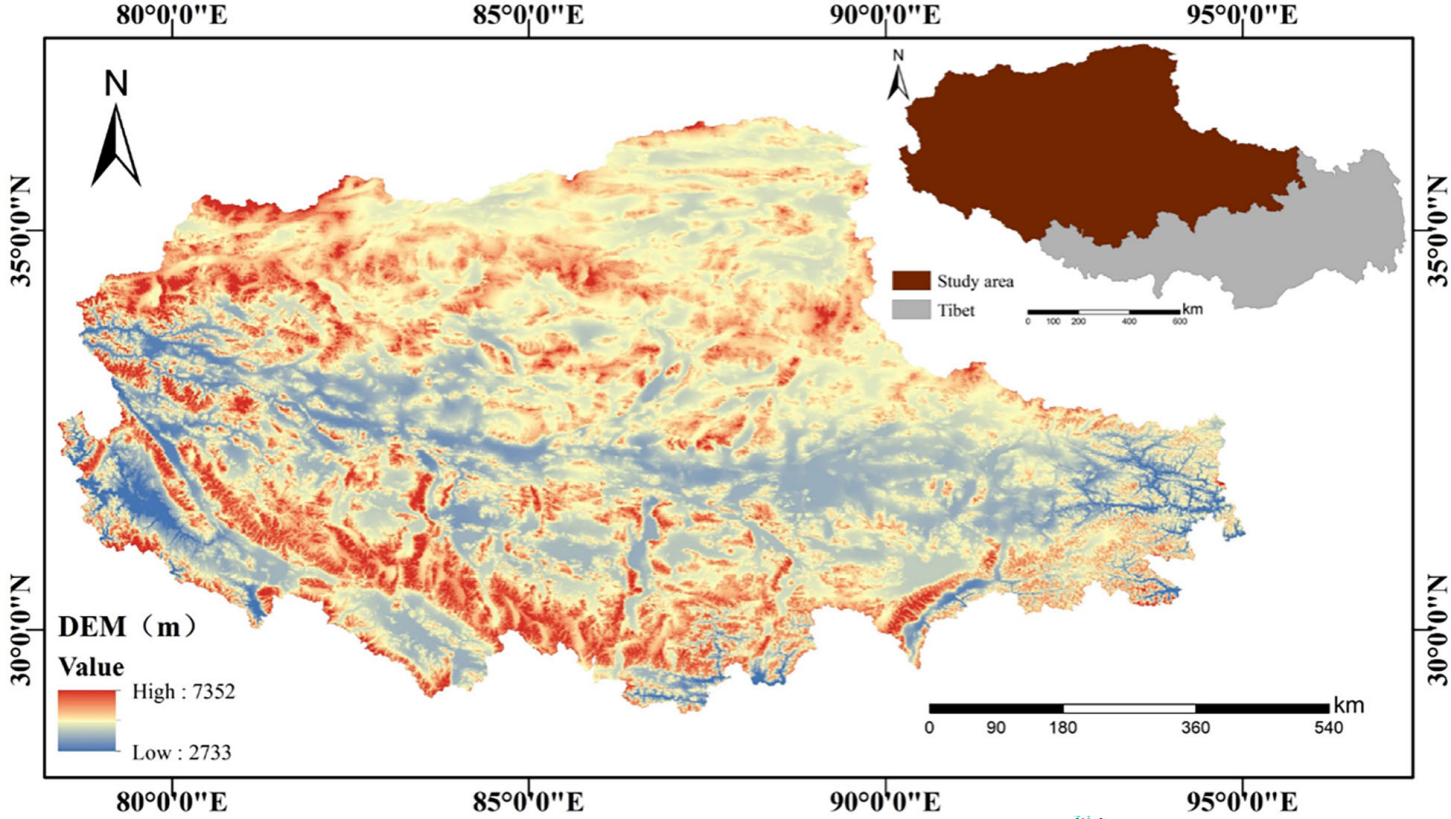
Location of the study area.

The northern Tibetan Plateau (29°56′N-36°30′N, 75°03′E-95°03′E), located in the hinterland of the Tibetan Plateau, covers an area of 4.46×10^5^km^2^ with an average elevation of about 4000 meters (Zhang et al., 2020) (**Figure 1**). The annual mean temperature ranges from -2.4°C to 1.6°C, decreasing from southeast to northwest. The average temperature of the coldest month (January) is -10~-12°C, and the average temperature of the hottest month (July) is 7-12°C. The annual precipitation is 66.3~894.5mm, and the annual evaporation is 1500~2300mm. The area of alpine grassland in the northern Tibetan Plateau is 4.21×10^5^km^2^, accounting for 94.4% of the total area. The grassland types mainly include alpine meadow, alpine grassland, and alpine desert grassland (Duan et al., 2019). The northern Tibetan Plateau habitats various large wild ungulates, with thousands of Tibetan antelopes, Tibetan gazelles, Tibetan wild donkeys, wild yaks, and rock sheep, among which Tibetan antelope, Tibetan wild donkey, and wild yak are unique species on the Qinghai-Tibet Plateau (Dawa, 2010). Grazing is the main human activity and has caused serious grassland degradation.

### 2.2 Data sources

Historical land use (1990 and 2015) was downloaded from the Data Centre for Resources and Environmental Sciences, Chinese Academy of Sciences (https://www.resdc.cn/). According to the specific situation and principal purposes of our study, the land uses were divided into six types including forest, grassland, farmland, water, barren, and urban. Future land-use scenarios of 2050 and 2100 were used in this study and the data was acquired from Li et al. (2017). Digital Elevation Model (DEM) data (90m resolution) were downloaded from the Geospatial Data Cloud website (http://www.gscloud.cn/). Fencing data were obtained from the published literature on fencing studies in the northern Tibet Plateau (Yu et al., 2016). Grazing data were obtained from Sun et al. (2020b) (**Table 1**).

**Table 1:** Data sources.

Data	Source	Resolution
Land use	https://www.resdc.cn/	1km
DEM	http://www.gscloud.cn/	90m
Fencing	https://doi.org/10.3390/su8111162	–
Grazing	https://doi.org/10.1016/j.scitotenv.2020.140721	1km

### 2.3 Methods

**Figure 2:**
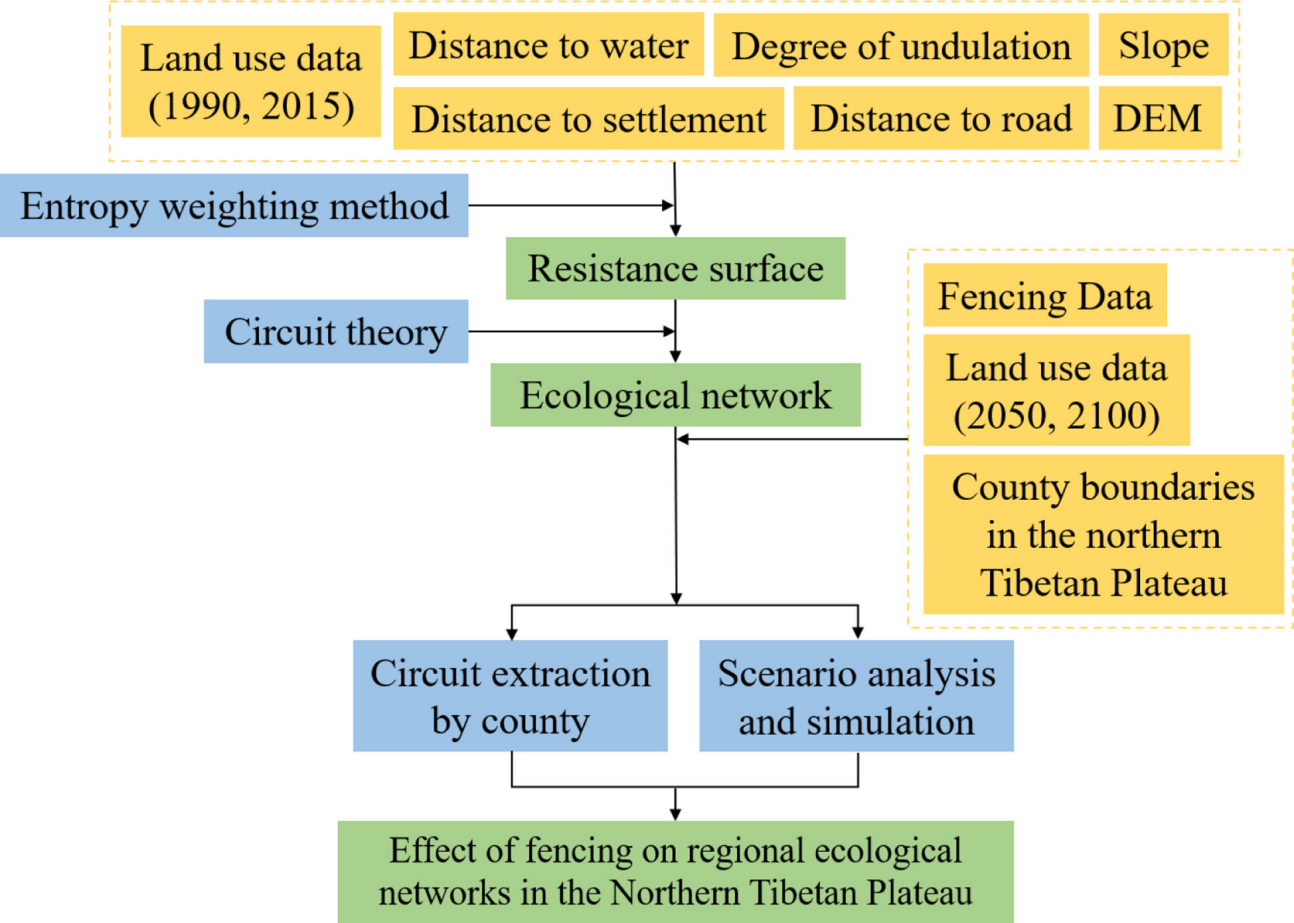
The working flowchart of this study.

To analyze the impact of fencing on the regional ecological network, we constructed the ecological network of the northern Tibetan Plateau using circuit theory under different scenarios. We set up different conditions as follows: no fencing in 1990, fencing in 2015, and unchanged fencing measures in 2050 and 2100 but with four land use scenarios. Based on the animal behaviors and migration activities of large wild ungulates in the study area, we selected different factors to model the ecological network and compare the impact of fencing on the ecological network at the regional scale, thus forming a general understanding of regional ecological network integrity (Huo et al., 2022). **Figure 2** showed the research working flowchart.

#### 2.3.1 Construction of resistance surface

In the study area, various landscape factors will hinder the migration of species to different degrees, so each environmental factor corresponds to different resistance values. The resistance value of each environmental factor refers to the suitability degree to which species can cross the surface of the environment. The ecological process of species movement in horizontal space and the flow and transmission of ecological functions are mainly affected by the land cover state and the degree of human disturbance, and because of the multi-plateau terrain conditions in the study area, elevation and slope also have resistance effects. Therefore, in this study, land use type, elevation and slope factors and human factors were used as influencing factors to construct resistance surfaces. In this study, based on the obtained shapefile format data of environmental factors in the northern Tibet Plateau, ArcGIS software was used to preprocess the basic data. Slope information of the study area was extracted from elevation data, and the Euclidean distance from the residential area, water area, and road was calculated by the distance calculation tool. Based on the actual situation in the study area and concerning existing studies, the resistance level of each landscape factor was classified into five levels (Ding et al., 2021; Yang et al., 2021). We assigned weights to each factor based on the entropy weighting method (Ji et al., 2020), and used the raster calculator in ArcGIS software, to overlay and obtain a resistance raster for the study area. Entropy is a measure of the disorder degree of the system. The smaller the entropy, the greater the effective information. The greater the entropy, the smaller the effective information. The entropy weight decision method is used to calculate the weight of each evaluation index, that is, the effective information of the evaluation index is used to calculate. The greater the effective information is, the greater the weight will be. Firstly, we standardized the data of each resistance factor, solved the correlation coefficient matrix, finally obtained the score of each factor, and weighted each resistance factor. The detailed resistance levels and weight assignments were shown in **Table 2**.

**Table 2:** Resistance factor ranking and weights.

Resistance	Weights	Ranking				
		1	2	3	4	5
Distance to road	0.07	>1500	1000-1500	600-1000	300-600	<300
DEM	0.12	<3200	3200-4100	4100-4700	4700-5100	>5100
Slope	0.17	<5	5-10	10-20	20-30	>30
Distance to settlement	0.12	>8000	3000-8000	1500-3000	500-15000	>500
Topographic relief	0.21	<100	100-200	200-400	400-700	>700
Land use	0.14	Water, forest	Grassland	Farmland	Barren	Urban
Distance to water	0.17	<800	800-1500	1500-2500	2500-4500	>4500

#### 2.3.2 Construction of ecological network based on circuit theory

In this study, we used Circuitscape 4.0 software to construct the ecological network. A buffer zone was first set up with a width of about 20% of the study area, from which 15 patches were randomly selected as habitat patches. Subsequently, the resistance surface was merged with the buffer zone, and finally, the combined resistance surface and the randomly selected patches were input into the software for calculation (Koen et al., 2014). The pairwise mode and the eight-neighborhood method were used to match the patches which were selected randomly in pairs and input into the Circuitscape software to perform the calculation (McLean and Rubio, 2022). After the generation of the current density map, the pre-established buffer area outside the study area is clipped to focus on the analysis of the currents within the study area. The final obtained clipped current density map can be used to describe the distribution pattern of the ecological network in the study area, from which information on the areas of high current density can be obtained. Moreover, valid information such as the maximum, average, and cumulative current density values in the area can be calculated to indicate the strength of network connectivity and the ease of migratory movements of wildlife in the study area (Fullman et al., 2020).

#### 2.3.3 Scenario analysis and simulation

Four land-use scenarios in 2050 and 2100 were selected in this study according to the study of Li et al. (2017). Scenario A1B describes rapid economic growth, rapid introduction of advanced technologies, and a balance between various energy sources. Scenario A2 describes continuous population growth, mainly regional economic development, intermittent per capita economic growth, and technological development. Scenario B1 has the same global population as scenario A1, with an emphasis on global economic, social, and environmental sustainability. Scenario B2 emphasizes local economic, social and environmental sustainability and focuses on local and regional levels (Li et al., 2017).

The current distribution in the study area was extracted by the mask extraction tool and calculated by Zonal statistics at county scale. We derived the maximum current, average current, and cumulative current density under different conditions. The conditions were set up as follows: no fences were set up in 1990, fences were set up in 2015, and unchanged fencing measures in 2050 and 2100 with the above four scenarios. The Origin software (OriginLab Corporation www.OriginLab.com) was used to draw the schematic diagram of the cumulative current density change and conduct a comparative analysis of scenarios (An et al., 2021).

## 3 Results

### 3.1 Fencing distribution and land-use change in the northern Tibetan Plateau

#### 3.1.1 Fencing distribution and grazing intensity at the county scale

**Figure 3:**
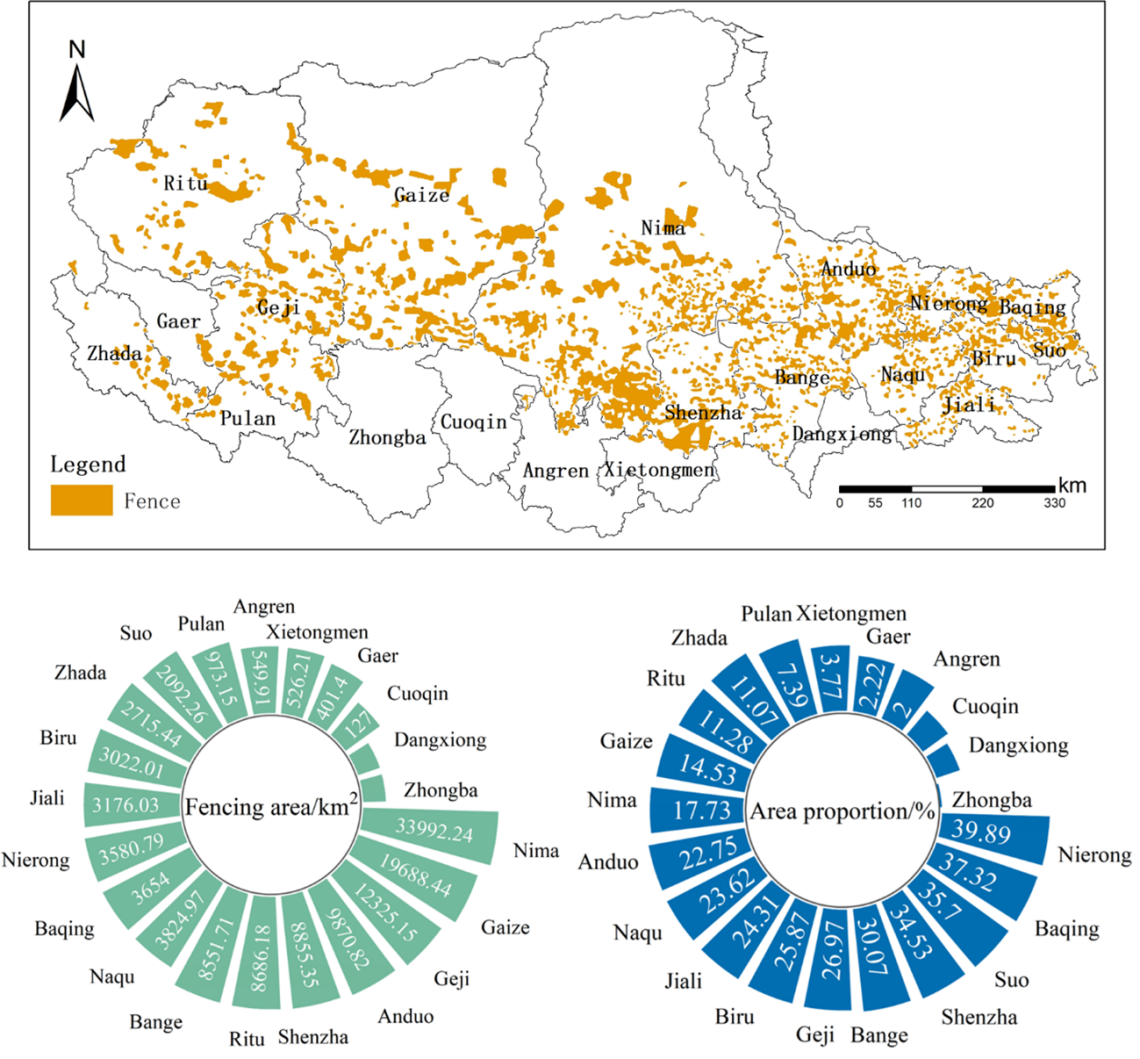
Fencing area and proportion by county.

**Figure 4:**
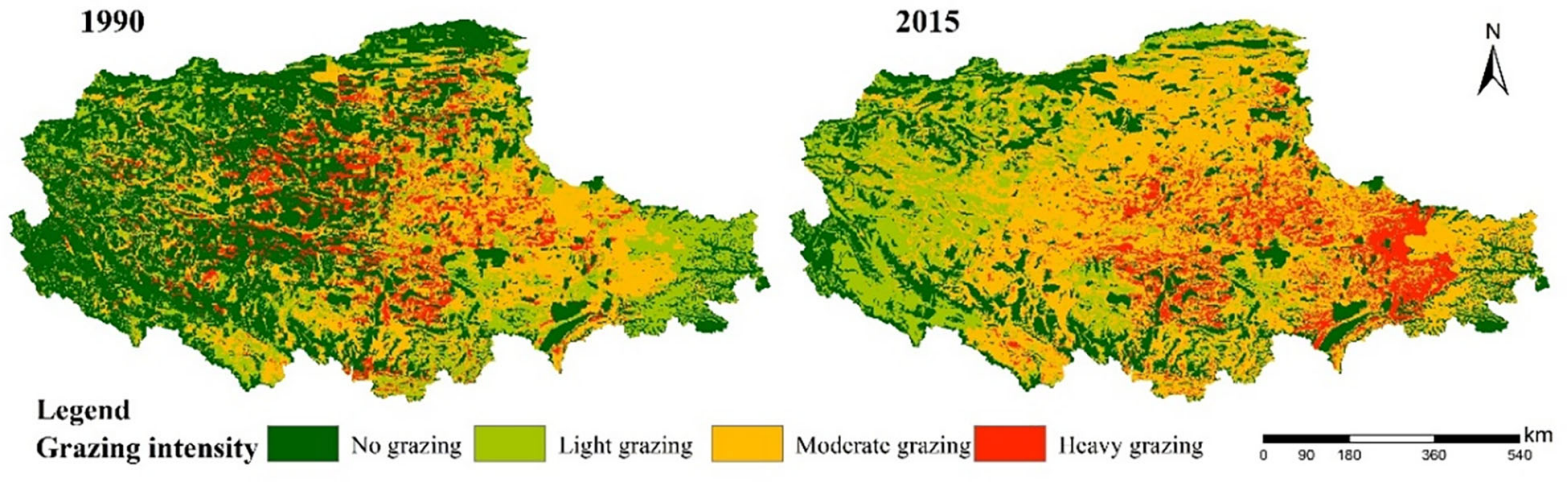
Grazing intensity in 1990 and 2015.

The construction of fences in the northern Tibet Plateau involved 21 counties, while the density of fences varies between different counties. The total area of fences was about 127,970 km^2^. The area of fencing within each county and its area proportion in each county were shown in **Figure 3**. As can be seen that Nima county had the largest fence area with 33992km^2^, followed by Gaizei county and Geji county, among which Dangxiong county and Zhongba county had the smallest fence area. Nierong county had the largest proportion of fencing area with 39.89%, followed by Baqing county, Suo county, Shenzha county, and Bange county. The proportion of fencing area in these counties was more than 30% of the total area of the county. Moreover, Pulan county, Xetongmen county, Gaer county, Angren county, Cuoqin county, Dangxiong county, and Zhongba county, these counties were less than 8% of the fencing area. **Figure 4** showed that the grazing intensity significantly increased from 1990 to 2015, with the grazing area shifting toward the southeastern part of the northern Tibetan plateau.

#### 3.1.2 Land use change analysis

**Figure 5:**
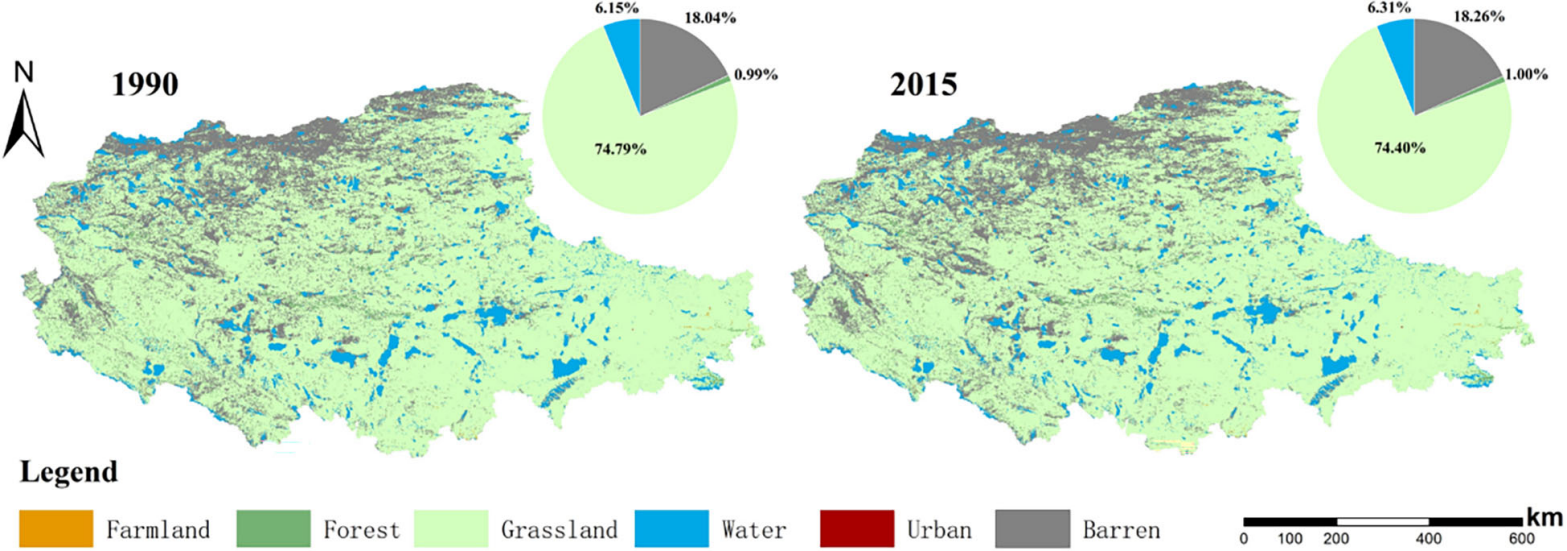
Land use in 1990 and 2015.

The land use pattern of the northern Tibetan Plateau in 1990 and 2015 is shown in **Figure 5**. The land use proportions of the northern Tibetan Plateau from high to low followed grassland, barren, water, forest, farmland, and urban. The area proportion of grassland was over 70%, while the area proportions of forest land, arable land, and urban land were low at less than 1%.

**Figure 6:**
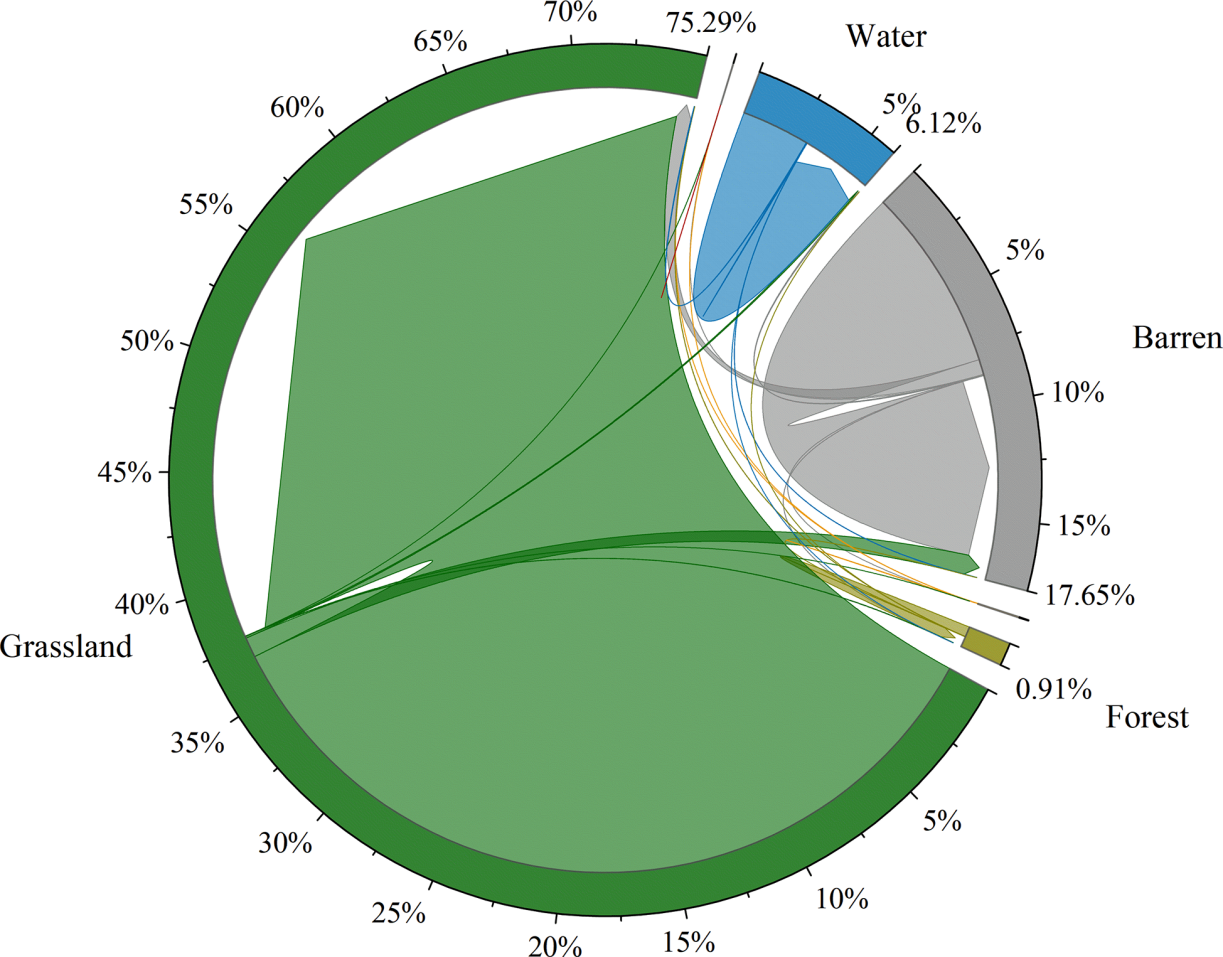
The land use transformation from 1990 to 2015.

Based on the land use data, we analyzed the land use changes from 1990 to 2015 (**Figure 6**). Affected by human activities and climate change, the grassland area decreased by a total of 4108km^2^, most of which was converted into the barren land, and the conversion proportion was as high as 90.84%. Meanwhile, the grassland degradation in the northern Tibetan Plateau is serious, concentrated in the northwestern part of the study area, including the northern part of Changtse and Nima counties, as well as Ritu, Geji, Kargil, and Zada counties. A rapid expansion of urban land has been observed, and the urban land in 2015 was 2.38 times that in 1990.

### 3.2 Ecological network changes in the northern Tibetan plateau

#### 3.2.1 Impact of land-use change on ecological networks

**Figure 7:**
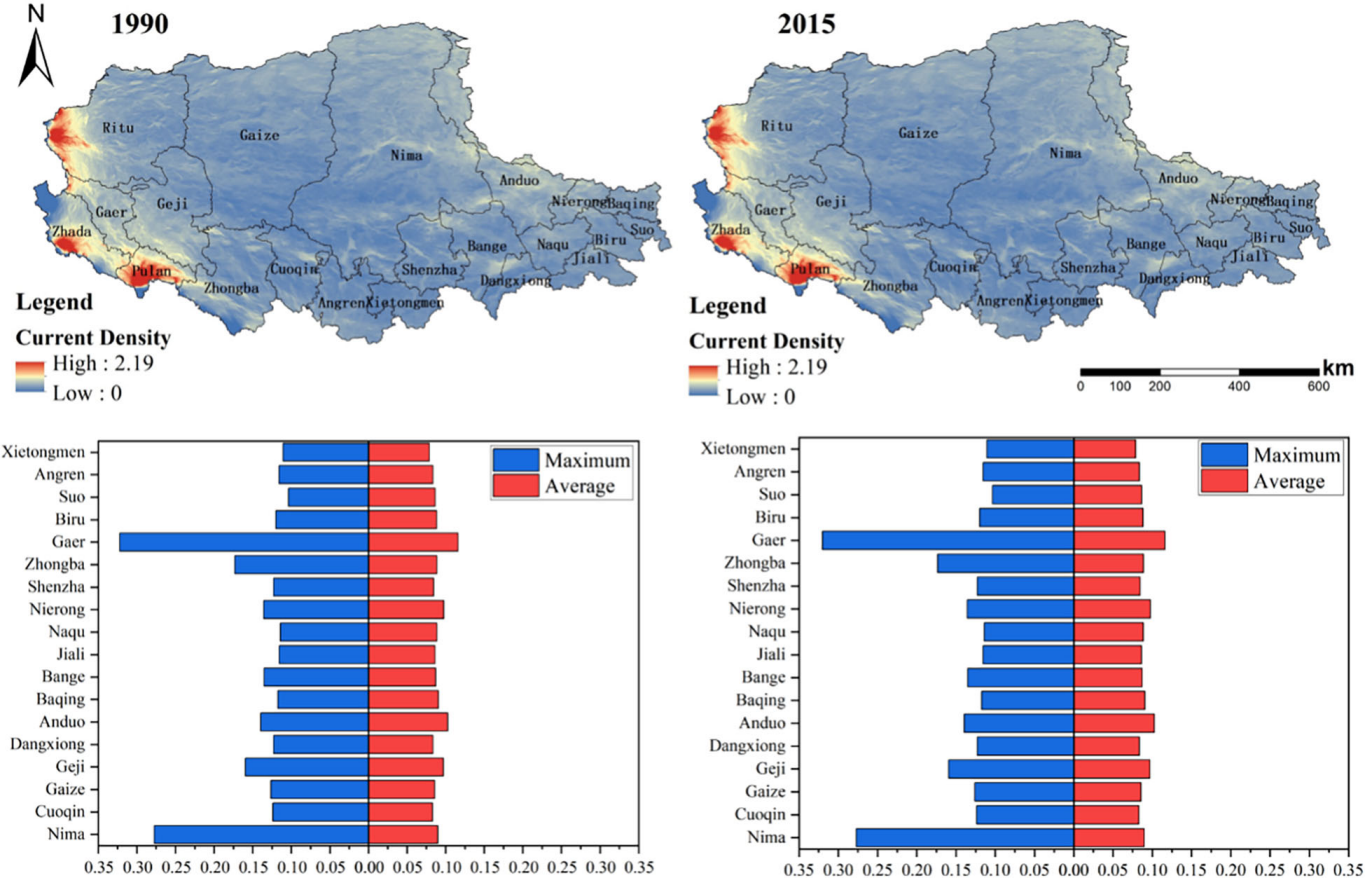
Current distribution, maximum and average value of current in each county of northern Tibet Plateau in 1990 and 2015.

**Figure 8:**
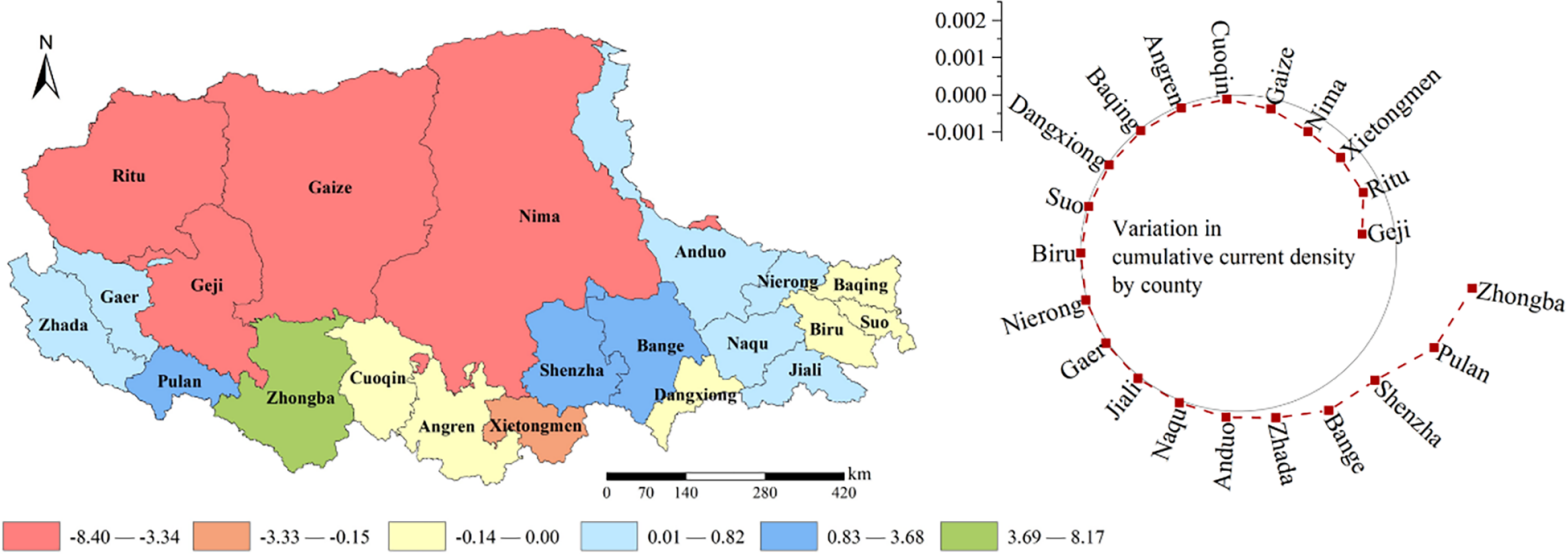
Variations of the cumulative current density in each county from 1990 to 2015.

We obtained the distribution of the current values of the ecological network of the study area in 1990 and 2015. Then the maximum and average values of current density together with the changes in cumulative current density in each county were compared for the two periods. **Figure 7** showed the current distribution for the maximum and average statistics of current density in 1990 and 2015 at the county scale. As shown in **Figure 8**, from 1990 to 2015, with grassland degradation, human activity intensification, and global climate change, more than 50% of the counties showed a decreasing trend in cumulative current density, which would lead to a decrease in landscape connectivity, and deteriorating integrity of the ecological network in the region. Among them, the counties with serious current density loss were distributed in the northwest and southeast, such as Jiaze County and Nima County, which were related to the serious grassland degradation and the intensification of grazing intensity in the corresponding areas.

#### 3.2.2 Impact of fencing construction on ecological network

**Figure 9:**
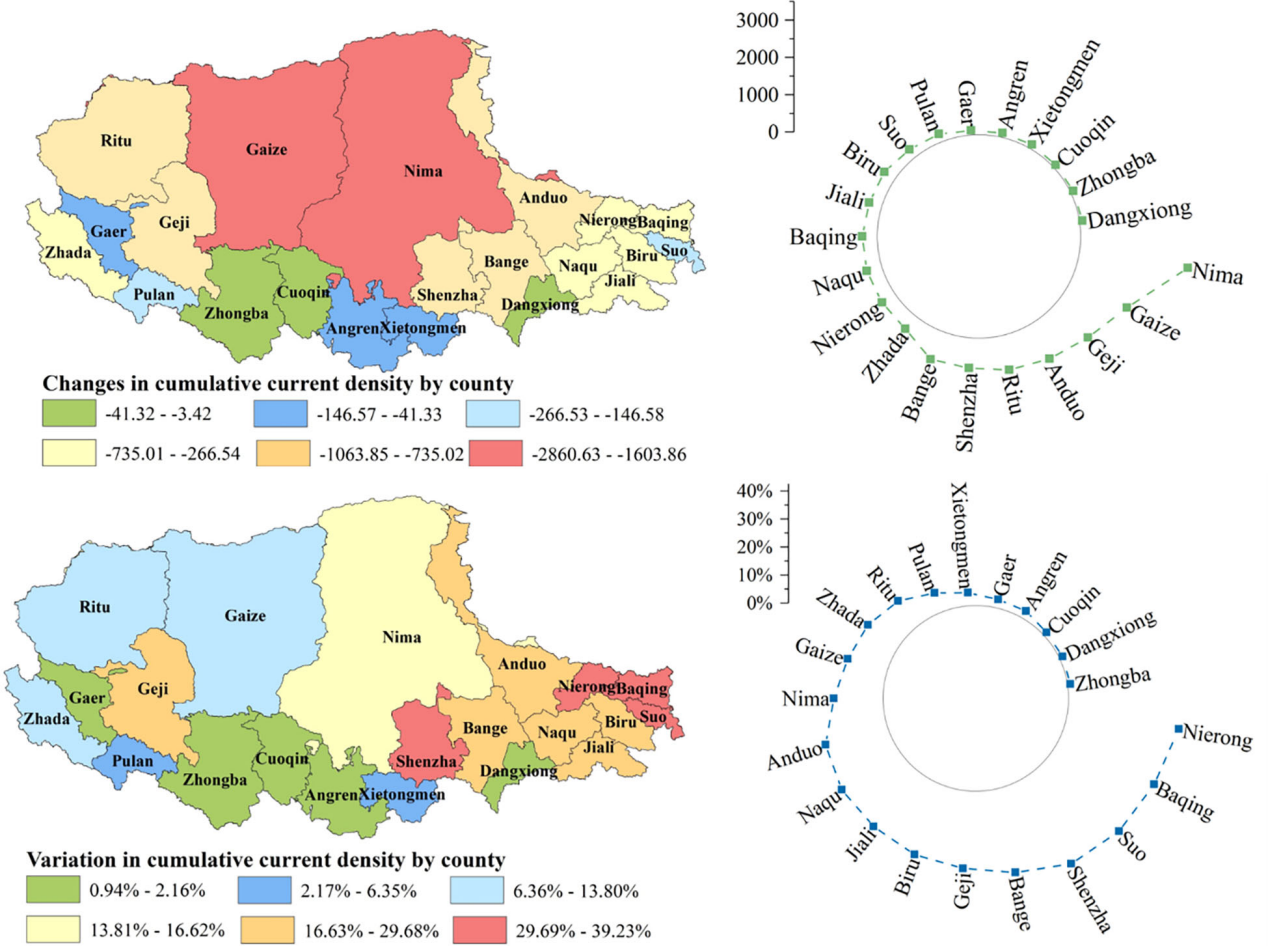
Changes in cumulative current density in each county due to fencing measurement.

As large-scale fence construction on the northern Tibetan Plateau had been carried out since 2004, we compared the cumulative current density changes before and after the establishment of the fence, thus quantifying the impact of the fence at county scale. **Figure 9** showed the status of cumulative current density loss occurring in each county after the construction of the fence. It can be seen that the counties with greater regional cumulative current density loss were mostly concentrated in the northwestern and eastern parts of the study area, which is related to the large area of degraded grassland and high grazing intensity in this region.

The results suggested that the fence construction caused the loss of cumulative current density within each county, making the network connectivity reduction and the ecological corridor number reduced. Moreover, the difference in the intensity of the fence made different rates of cumulative current density loss within each county.

#### 3.2.3 Changes in ecological networks under future land-use scenarios

**Figure 10:**
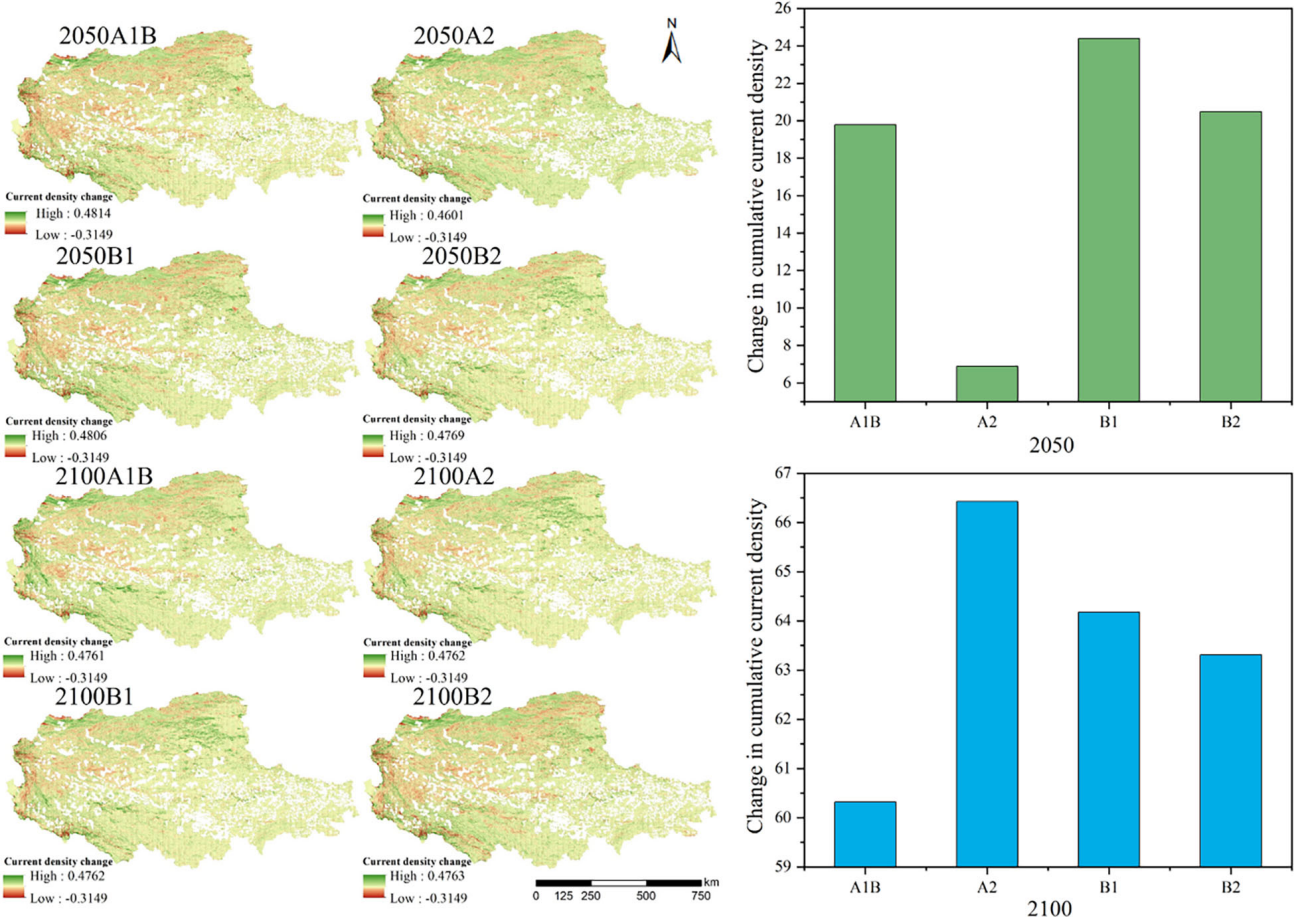
Cumulative current density changes under different future scenarios in the northern Tibetan Plateau.

Under the condition that the current fencing measures remain stable, ecological network levels under four different land use scenarios in 2050 and 2100 were compared. The land use scenarios considered economic and social development, the impact of human activities, and climate change. On this basis, the ecological network of the northern Tibetan Plateau was constructed by the circuit theory approach, and the current density distribution was obtained. **Figure 10** showed the comparison of the 8 current density distributions under different scenarios with the current density distribution after the fence construction in 2015. It was obtained that the network connectivity is better under the B1 scenario, which is related to the continued economic and social development, the active introduction of clean technologies, and the decreasing trend of the population under this scenario.

## 4 Discussion

### 4.1 Effect of fencing on regional ecological networks

Our study showed that the fencing measures not only protected grassland resources in the northern Tibetan Plateau, but also had negative effects on the regional ecological network, which reduced the landscape connectivity and network connectivity in the region and blocked the species’ interaction. That is, after the extensive establishment of fences in 2015, the current density loss in the study area had taken place, and the fence density variations caused the difference in the cumulative current density loss rate in each county. High-intensity fencing can significantly reduce the cumulative current density in the area, severely hindering the migration of wild ungulate species. Large area of fencing has now been built in Nima, Shenza, Ritu, and Gaize counties, et al., which harm wildlife movement. It has been reported that the fences affected the activities of pronghorn antelope (Flesch et al., 2010), and some herdsmen have found that wild donkeys, argyle sheep, and other species are entangled in the fence and killed. As shown in previous studies, fencing restricts the activity range of Tibetan antelope and is detrimental to their migratory activities (Arie et al., 2016). Each year in early summer, Tibetan antelopes migrate in herds to the north to lamb. Their migration routes are fixed and when they find obstacles along their traditional migration routes, they are unable to complete their migration (Jing et al., 2020). Extensive and long-distance fencing not only keeps wildlife out but also completely cuts them off from drinking water and grazing. According to previous studies, the north side of Dazicuo, on the border of Nima county and the Shuanghu county, is completely fenced off, preventing wildlife from drinking from the lake. Thus, fencing directly affects the migratory movements of wildlife, with more fencing cutting off more migratory routes (Wu et al., 2021). In the long run, these fences would divide the intact and interconnected ecosystem into fragmented patches, leading to decreased regional grassland connectivity and wildlife habitat fragmentation, potentially leading to species extinction (Sun et al., 2020a). This may lead to a loss of biodiversity and ultimately cause degradation of the ecosystem after long-term fencing.

### 4.2 Effect of land use on regional ecological network

Land use is always changing due to urbanization, grazing activities, and global climate change (Zhang et al., 2021). However, changes in land use structure, such as grassland degradation and urban expansion, will lead to intensified habitat fragmentation and block the flow of energy information exchange in the regional networks, which is not conducive to regional ecological sustainable development (Li et al., 2022). According to the research of Wu et al. (2015), land use change makes species’ ecological network break seriously, and quality decline. Therefore, the study of the impact of land-use change on the ecological network is conducive to the restoration and improvement of regional ecological networks and deepens the understanding of the ecological network from the perspective of the ecological process.

As shown in our study, given the classification criteria of grazing intensity in the existing studies, the grazing intensity of the northern Tibetan Plateau was divided into four classes of areas, no grazing area, light grazing area, moderate grazing area, and heavy grazing area (Jin et al., 2020; Hu et al., 2022). We found that the degree of grazing significantly intensified from 1990 to 2015, and the grazing area shifted to the southeastern part of the northern Tibetan Plateau. Climate change and anthropogenic disturbances have had an irreversible impact on grassland degradation in the northern Tibetan Plateau (Yang et al., 2020), where the degradation of grasslands and human activities have led to a decrease in the structure and function of ecological networks. Moreover, comparing the ecological network under the four future scenarios, we found that network connectivity would improve under the scenario of coordinated economic, social, and environmental development, which is consistent with previous studies. Based on the study of Wang et al. (2022), ecological conservation scenarios can effectively protect landscape connectivity. Therefore, in future development, it is necessary to reduce the change in land use caused by human factors, so as to reduce the deterioration of ecological network connectivity caused by land use change.

### 4.3 Limitations and future work

This study analyzed the effects of fencing on regional ecological network of the northern Tibetan Plateau. Thus, the results of the study can provide references for grassland restoration and the protection of wildlife. However, there were still some limitations, which can be concluded into the following points: 1) There are still few studies on the ecological effects of large-scale fencing, especially on the Qinghai-Tibet Plateau. Also, the availability of fencing data may influence the results. Due to the incomplete statistical nature of the fence distribution, the ecological network constructed based on the fence data in this study and the result statistics may have some deviations. 2) Since the scenario analysis selected in this study is rather homogeneous at present, not considering climate change, changes in species habitat, integrated measures, etc. (Wu et al., 2022), and also lacking some empirical data, the results of this study may have some uncertainties. Therefore, considering the above limitations, in future research, we will integrate more drivers and collect more comprehensive data to make the results more reliable and reduce the interference of uncontrollable factors, which will lead to more accurate and credible analysis results. 3) Since there is no field survey in the study area, the results are uncertain to some extent. In the follow-up research, we will add a field sampling survey to verify our results.

## 5 Conclusions

In this study, to investigate the influence of fencing on the regional ecological network of the northern Tibetan Plateau, we constructed the ecological network based on the circuit theory method, which was extracted and analyzed at county scale. We found that the grassland in the northern Tibet Plateau has been degraded because of anthropogenic factors such as grazing and urban expansion, which led to a decrease in the connectivity of ecological networks within the region. In order to solve the problem of grassland degradation, a large scale fence has been built on the northern Tibetan plateau. However, the establishment of fences can reduce landscape connectivity and network connectivity in the study region, which can act as barriers to wildlife migration. Especially in the northwest and east of the study area, the current density at the county scale was greatly lost due to the high-intensity fence construction. The results of the study have important ecological significance for developing reasonable conservation measures for grassland restoration, protecting wildlife, and maintaining regional ecological balance.

## Data availability statement

The original contributions presented in the study are included in the article/supplementary material. Further inquiries can be directed to the corresponding author.

## Author contributions

YZ: Data curation, Writing- Original draft preparation, Software, Validation; SL: Supervision, Writing- Reviewing and Editing. YD: Data curation, Methodology, Software. FW: Methodology, Software, Investigation. HL: Data curation, Writing- Original draft preparation, Software, Validation. YL: Methodology, Software, Visualization. All authors contributed to the article and approved the submitted version.
